# Neutrophils in colorectal cancer: mechanisms, prognostic value, and therapeutic implications

**DOI:** 10.3389/fimmu.2025.1538635

**Published:** 2025-02-28

**Authors:** Xingyue Wang, Shukang He, Xiangmei Gong, Shijun Lei, Qianwen Zhang, Junqi Xiong, Yang Liu

**Affiliations:** Department of Clinical Laboratory, Union Hospital, Tongji Medical College, Huazhong University of Science and Technology, Wuhan, China

**Keywords:** neutrophils, colorectal cancer, gut microbiota, tumor microenvironment, therapies, prognosis

## Abstract

Neutrophils, the most abundant myeloid cells in human peripheral blood, serve as the first defense line against infection and are also significantly involved in the initiation and progression of cancer. In colorectal cancer (CRC), neutrophils exhibit a dual function by promoting tumor events and exerting antitumor activity, which is related to the heterogeneity of neutrophils. The neutrophil extracellular traps (NETs), gut microbiota, and various cells within the tumor microenvironment (TME) are involved in shaping the heterogeneous function of neutrophils. This article provides an updated overview of the complex functions and underlying mechanisms of neutrophils in CRC and their pivotal role in guiding prognosis assessment and therapeutic strategies, aiming to offer novel insights into neutrophil-associated treatment approaches for CRC.

## Introduction

Colorectal cancer (CRC) is among the most prevalent malignant tumors worldwide, with its incidence ranking fourth and mortality ranking third, according to the latest global cancer data (1). The CRC encompasses hereditary, sporadic, and colitis-associated colorectal cancer (CAC) forms (2). Indeed, extensive epidemiological and experimental studies have consistently revealed that chronic inflammation plays a pivotal role in the initiation and progression of CRC (3–6). Depending on the stage at which inflammation affects CRC development, it can be categorized into three types: pre-tumor chronic inflammation, tumor-triggered inflammation, and treatment-induced inflammation. These processes activate tumor-promoting innate immune cells, inhibit anti-tumor adaptive immune cells, and establish an immunosuppressive tumor microenvironment (TME) (5).

Neutrophils serve as the primary line of defense against microbial infection by employing various mechanisms: (1) phagocytosis, wherein they engulf pathogenic microorganisms to form phagocytic vacuoles and generate toxic chemicals for microbial eradication (7); (2) degranulation, involving the controlled release of cytotoxic enzymes and proteases from intracellular vesicles to eliminate microorganisms (8); and (3) the formation of neutrophil extracellular traps (NETs), which are protein-DNA structures that ensnare and neutralize microorganisms (9). These processes encompass both oxidative and non-oxidative pathways that drive neutrophils to exert their antimicrobial effects with potent killing capabilities (10, 11). It has been traditionally believed that circulating neutrophils cannot proliferate after reaching maturity, leading to rapid depletion under steady-state conditions with a half-life of less than 1 day (12). However, it is important to note that neutrophils exhibit high plasticity and can transition into different functional states in various disease contexts, consequently impacting their lifespan (13). Recent studies have demonstrated that neutrophils recruited to tissues can undergo reverse migration back into the bloodstream after functioning and can even migrate to distant organs to induce inflammatory responses (14, 15).

Neutrophils not only play a crucial role in the body’s defense against infection, but also exert significant influence in tissue repair, autoimmune diseases, and tumor development (16–18). Due to the heterogeneity and plasticity of the tumor-associated neutrophils (TANs) within the TME, the precise subtype characterization of TAN has become a crucial research focus. Neutrophils can be polarized into TAN1 or TAN2 subtypes in the TME. TAN1 exhibits increased expression of immune-activating cytokines and chemokines, along with reduced arginase. While TAN2 demonstrates elevated expression of angiogenic factors, stroma-degrading enzymes, and substantial arginase, exerting immunosuppressive effects (19). The absence of widely recognized subgroup markers has hindered comprehension of neutrophil heterogeneity. Consequently, higher-dimensional techniques are needed for investigation. A study employed time-of-flight mass spectrometry to identify seven neutrophil clusters in human melanoma: the CD117^+^CD66b^+^ progenitor population and Cneut1 to Cneut6. It shows the heterogeneity in neutrophil differentiation. Notably, Cneut2 has the highest expression of CD101, CD10, and CD16, indicating its maturity (20). Furthermore, recent advancements in single-cell RNA sequencing (scRNA-seq) analysis have identified distinct TAN subsets in cancers. The four TAN subgroups in pancreatic ductal adenocarcinoma are designated as TAN1 to TAN4. TAN1 is characterized by elevated expression of VEGFA, PLAU, and LGALS3. TAN2 expresses inflammation-related genes NLRP3 and PDE4B. TAN3 is a transitional stage with high expression of transendothelial migration genes. TAN4 preferentially expresses interferon-stimulated genes, such as IFIT1, IFIT2, and ISG1 (21). The six TAN subgroups identified in primary liver cancer are Neu_01_MMP8, Neu_07_APOA2, Neu_08_CD74, Neu_09_IFIT1, Neu_10_SPP1, and Neu_11_CCL4. Subgroups Neu_09/10/11 are associated with a poorer prognosis. The expression of CD274, encoding PD-L1, is elevated in TANs, highest in the Neu_09_IFIT1 subgroup (22). To further investigate the tissue and phenotype plasticity of neutrophils, researchers integrated transcriptome data from 225 samples across 17 cancers. The analysis revealed substantial heterogeneity, characterized by ten distinct cell states, including S100A12^+^, HLA-DR^+^CD74^+^, VEGFA^+^SPP1^+^, TXNIP^+^, CXCL8^+^IL1B^+^, CXCR2^+^, IFIT1^+^, ISG15^+^, MMP9^+^, NFKBIZ^+^, HIF1A^+^, and ARG1^+^ neutrophils (23).

All these indicate that neutrophils are not single-function cells with terminal differentiation, but rather their differentiation and maturation trajectories can be significantly altered under specific conditions, leading to diverse functional outcomes. Here, we discuss neutrophils’ multifaceted contributions to CRC development, encompassing NET formation, interactions with gut microbiota, and intricate crosstalk within the TME. Furthermore, we discuss the inhibitory effect of neutrophils on CRC progression and their potential to guide the prognosis and treatment of CRC. We hope to provide novel perspectives for optimizing CRC treatment strategies.

## Methods

### Literature search

Two independent researchers conducted a comprehensive search to identify the studies published in databases, such as PubMed and Embase, from January 1st, 2000, to November 30th, 2024. The following medical subject headings (MeSH) or keywords were used: “colorectal cancer,” or “colorectal carcinoma,” or “colon cancer,” or “colon carcinoma,” or “rectum cancer,” or “rectum carcinoma,” or “CRC,” and “neutrophil,” or “TAN,” or “inflammation”.

### Selection criteria

The inclusion criteria were as follows: (1) original experimental studies on the roles of neutrophils in the development of CRC, or sequencing analysis of neutrophils in CRC, or bioinformatics analysis, or meta-analysis. (2) full text in English; (3) studies with necessary ethical approvals.

The exclusion criteria were as follows: (1) studies involving human or animal subjects without ethical approval; (2) expert opinions or comments.

The above methods include the literature required for the main body of the review. In addition to CRC, we also referred to some relevant studies on other cancers to further discuss the function of neutrophils.

## Neutrophil recruitment in CRC

The classical process of intravascular neutrophil recruitment during inflammation involves five coordinated stages: tethering, rolling, adhesion, crawling, and transmigration (24). The entire process is regulated by a variety of inflammatory-related molecules, including chemoattractant subfamilies (e.g., chemokines and their receptors, chemotactic lipids, complement anaphylatoxins), cytokines, integrins, and cell adhesion molecules (24–26). In addition to the classical molecules mentioned above, a recent study discovered that neutrophil surface RNAs with glycan modifications facilitate neutrophil recruitment and promote neutrophil-endothelial interactions by binding to P-selectin on endothelial cells (27).

Tumor progression is always regarded as a chronic inflammatory process (28, 29), during which the recruitment pattern of neutrophils is consistent with physiological inflammation. Chemotactic compositions present in the TME mainly include CXCL8 family chemokines (e.g., CXCL1/2/3/5/6/7/8) (30), cytokine interleukin (e.g. IL-1β, IL-8, IL-17) (31–33), and the associated signaling pathways (e.g., TGF-β/Smad3) (34), responsible for neutrophil recruitment and polarization. In CRC, chemokines (e.g., CXCL1/2) and cytokines (e.g., IL-8, and TGFβ) (35–37) are also involved in regulating neutrophil enrichment to the TME and facilitating their polarization towards TAN2 phenotype, while IL-22 plays a role in recruiting beneficial neutrophils (38).

## Protumoral role of neutrophils in CRC

Neutrophils are increased in peripheral blood and tumors of patients with CRC, as compared to healthy donor peripheral blood and paired adjacent non-tumor colon tissue, and are associated with higher cancer stage and histological grade (39). The phenomenon has been found that ulcerative colitis patients with more neutrophil infiltration are at a higher risk of developing CAC (40). In both colitis and CAC models, neutrophil infiltration increased as the disease progressed, and neutrophils may promote the transition from colitis to CAC (40). The above studies suggest neutrophils are important in the development and progression of CRC.

### NETs

**Figure 1:**
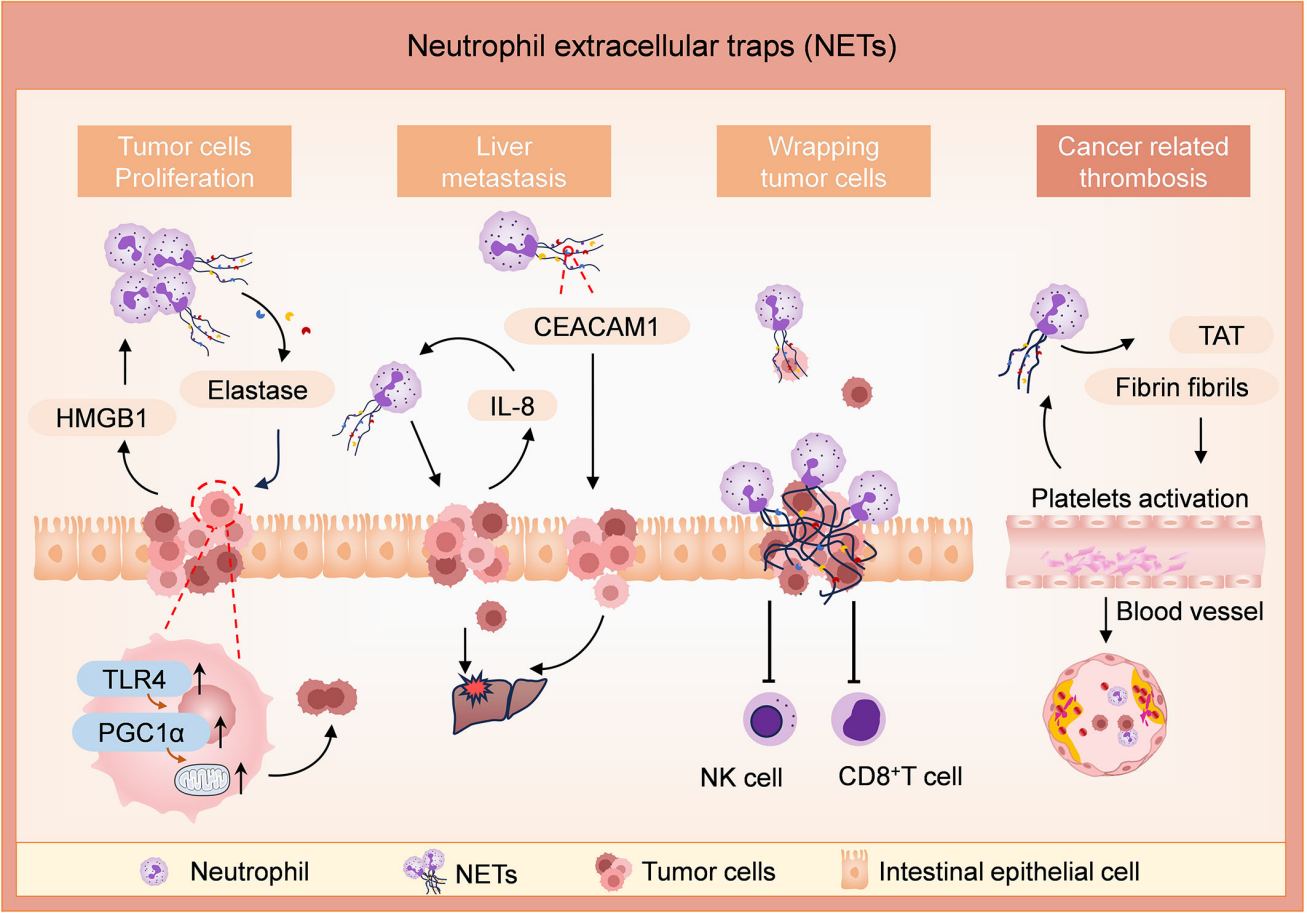
Roles of NETs in tumor development, metastasis, and thrombogenesis in CRC. Levels of NETs are enhanced by tumor-derived damage-associated molecular pattern proteins (such as HMGB1). NETs release elastase and promote tumor cell proliferation by enhancing their mitochondrial biogenesis in the TLR4/PGC1α-pathway. In addition, NETs enhance liver metastases in an IL-8 or CEACAM1-dependent way. The NET formation is enhanced by tumor cells-derived IL-8. CEACAM1, anchored on NETs, facilitates the adhesion and migration of tumor cells by weakening endothelial connections. NETs create an immunosuppressive niche and protect tumor cells from cytotoxicity mediated by CD8^+^ T cells and NK cells. NETs induce thrombogenesis by increasing the expression of TAT complexes and fibrinogen fibrils, and the procoagulant environment enhances the levels of NETs in turn.

NETs are extracellular network structures consisting of decondensed chromatin, histones, and granzymes (41). NETs serve to capture and eliminate extracellular pathogens, thereby contributing to the body’s defense against infections (42). Emerging research indicates that NETs also play an important role in non-communicable diseases, encompassing autoimmune diseases, allergic diseases, blood clotting disorders and cancers (43). Notably, NETs possess potent tumorigenic properties and contribute to the initiation, spread, and thrombotic complications associated with cancer (44). An Ewing sarcoma study first demonstrated that activation of intratumor TANs resulted in NETs releasing, which was associated with a poor prognosis (45). NETs can capture circulating tumor cells and wake up tumor cells *in vivo* (46–49). Indeed, NETs promote cancer progression through direct promotion of cell growth and facilitating tumor metastasis in CRC (**Figure 1**).

NETs can directly modulate the metabolic program of tumor cells in murine models of metastatic CRC. Under a hypoxia state, tumor cells in metastatic CRC models release damage-associated molecular pattern proteins, such as HMGB1, which recruit neutrophils to the TME and trigger NET formation. Then, neutrophil elastase released by NETs activates TLR4 on tumor cells, leading to the upregulation of PGC1α, enhanced mitochondrial biogenesis, and accelerated tumor cell proliferation (50).

NETs promote liver metastasis in CRC *in vivo* and *in vitro*. A study showed that NETs were frequently detected in CRC tissue slices (37/85, 44%). It revealed a preferential localization of NETs in the central or front of invasion within CRC tissue sections and a significant correlation with tumor grade and lymph node metastasis. The purified NETs induce filamentous foot formation and cell migration in CRC cell lines, suggesting their potential to activate epithelial-mesenchymal transition-like processes and promote progression and metastasis in CRC (51). *In vivo* and *in vitro* investigations discovered that NETs promote liver metastasis in CRC by inducing the expression of IL-8 in tumor cells. Overexpression of IL-8 causes the activation of neutrophils to release more NETs, which further promotes liver metastasis (52). CEACAM1 is an important molecule on NETs, facilitating the adhesion and migration of colon tumor cells at metastatic sites by mediating the interaction between tumor cells and NETs, as well as weakening endothelial connections (53). NETs create an immunosuppressive niche to facilitate the growth and translocation of the tumor. NETs directly protect the tumor cells from the immune cells by shielding tumor cells from cytotoxicity mediated by CD8^+^ T cells and natural killer (NK) cells (54). In the mouse CRC model, NETs have been shown to cause dysfunction of T cell metabolism and function via the PD-1/PD-L1 signaling pathway, ultimately promoting tumor growth (55). Thus, NETs contribute to the development of CRC tumors in several ways.

Beyond the roles in the development and metastasis of CRC, NETs are involved in the formation of thrombin antithrombin (TAT) and blood vessels, thereby causing adverse clinical outcomes. There are few studies on how NETs affect angiogenesis in CRC. During angiogenesis, NETs support human endothelial cells’ proliferation and tubular capacity *in vitro (* 56, 57). NETs in CRC individuals significantly increase the formation of TAT complexes and fibrinogen fibril, inducing endothelial cells to exhibit a procoagulant phenotype, resulting in enhanced platelets procoagulant activity by inducing phosphatidylserine exposure to platelets (58). Furthermore, platelets from patients with CRC stimulate healthy neutrophils to produce NETs (58). Thus, inhibition of NET is an important strategy for preventing tumor-related thrombosis in CRC patients.

The majority of studies have highlighted the pro-tumor effects of NETs but also show negative effects during tumor chemotherapy. For instance, research has demonstrated that chemotherapy-induced IL-1β can trigger NET formation, which subsequently activates the TGF-β signaling pathway in tumor cells, promoting epithelial-mesenchymal transition and chemotherapy resistance in breast cancer lung metastasis (59). However, a recent study has revealed an unexpected outcome: chemotherapy-induced NETs can inhibit CRC tumor growth. The combination of the glutaminase inhibitor CB-839 and 5-fluorouracil induces IL-8 expression in PiK3CA-mutated CRC cells, attracting neutrophils to tumor tissues, inducing NET formation and releasing Cathepsin G. Cathepsin G enters tumor cells through the cell surface protein RAGE, leading to mitochondrial translocation of pro-apoptotic BAX and triggering apoptosis in tumor cells (60). This study significantly enhances our understanding of the complex roles of neutrophils and NETs in anti-cancer therapy.

### Crosstalk with gut microbiota

Previous studies have established a correlation between dysregulation of the gut microbiota and the development of CRC. The gut microbiota, closely associated with CRC, constitutes a crucial component of the TME. Certain bacteria, such as *Fusobacterium nucleatum* (*F. nucleatum*), *Escherichia coli*, and *Peptostreptococcus* spp, are presumed to be pro-carcinogenic in CRC and were found to be abundant in patients, while potentially protective bacteria, including *Roseburia*, *Clostridium*, and *Bifidobacterium* were decreased (4). Research has indicated that the gut microbiota can impact the development of CRC by releasing various metabolites, proteins, and macromolecules that interact with colon epithelial cells and immune cells (61).

**Figure 2:**
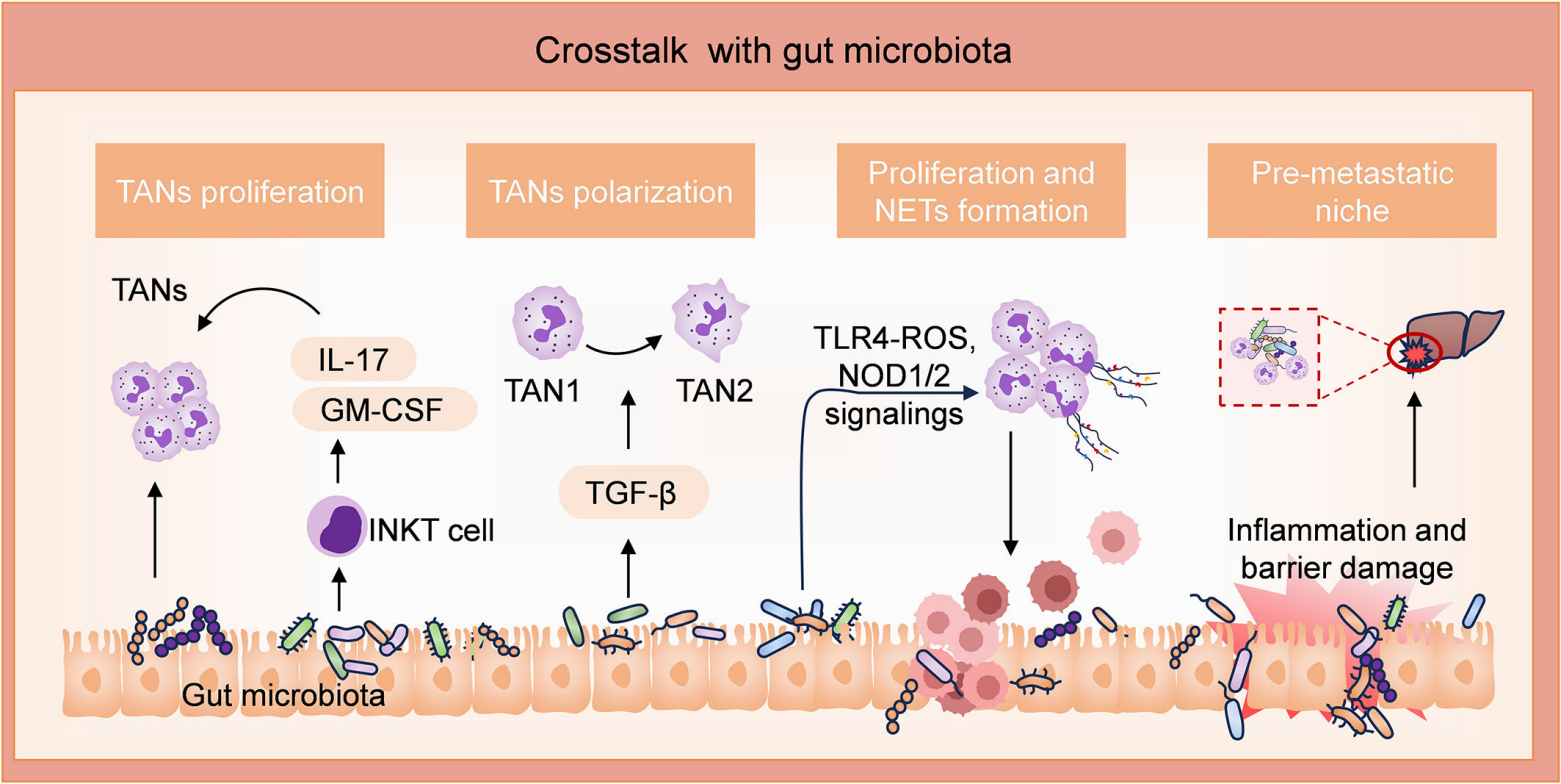
Involvements of neutrophils through interaction with gut microbiota in CRC. *Peptostreptococcus anaerobius* (*P. anaerobius*) and *Fusobacterium nucleatum* (*F. nucleatum*) possess pro-tumor activities. *P. anaerobius* promotes the proliferation of TANs. *F. nucleatum* enhances the expression of iNKT cell-derived IL-17 and GM-CSF, which further promotes the expansion of TANs. *F. nucleatum* also facilitates the differentiation of TANs to TAN2 by activating the TGF-β pathway. Furthermore, by activating TLR4-ROS and NOD1/2 signalings, *F. nucleatum* induces the formation and release of extensive NETs, thereby promoting tumor growth and metastasis. In addition, high levels of inflammation induced by the gut microbiota facilitate liver metastasis niche by compromising the intestinal barrier.

Neutrophils, as an integral part of immune cells, are known to play a significant role in the onset and progression of CRC through their interactions with the gut microbiota (**Figure 2**). Using fluorescence *in situ* hybridization and double RNA sequencing technique, researchers also discovered a novel association between *Bacteroides fragilis* and neutrophil infiltration in CRC (62). CRC is more prevalent in individuals with obesity (63, 64). Indeed, A recent study demonstrated an enrichment of neutrophils in visceral adipose tissue (VAT) and the presence of bacteria, primarily *Streptococcaceae* and *Ruminococaceae*, in the VAT of obese individuals. It has been revealed that obesity-induced translocation of gut microbiota leads to infiltration of neutrophils in VAT, followed by activation by bacteria or their metabolites, resulting in pro-inflammatory and anti-apoptotic functions (65). *Peptostreptococcus anaerobius* has been found to promote the development of CRC and modulate tumor immunity. In ApcMin^+/+^ mice treated with *Peptostreptococcus anaerobius*, myeloid suppressor cells, tumor-associated macrophages, and TANs were significantly expanded, which are linked to chronic inflammation and tumor progression (66). Invariant natural killer T cells (iNKTs) represent an evolutionarily conserved subset of lymphocytes situated at the interface between innate and adaptive immunity. The study revealed that tumor-infiltrating iNKTs promoted tumorigenesis in CRC patients. *F. nucleatum* can induce iNKTs cell-mediated recruitment of neutrophils, which inhibit the adaptive immune response in cancer, by expressing IL-17 and GM-CSF (67). *F. nucleatum* infection has been shown to activate TGF-β, a critical signaling pathway that regulates TAN differentiation (68). Current findings indicate that CRC with a high abundance of *F. nucleatum* have increased neutrophil counts and elevated levels of NETs. *F. nucleatum* induces the formation and release of extensive NETs by activating TLR4-ROS and NOD1/2 signaling, thereby promoting the growth and metastasis of CRC (69).

Bacteria translocation not only facilitates the proliferation of tumor cells at the primary site but also enhances tumor metastasis. Various bacterial strains have been demonstrated to activate innate and adaptive immune cells, thereby amplifying inflammation at the primary site and compromising intestinal barrier function (70). After damaging the barrier function of the gut, bacteria migrate from the lumen through the blood circulation to the liver, promoting the recruitment of neutrophils in metastatic lesions and establishing a pro-inflammatory immune microenvironment conducive to pre-metastatic niche formation in the liver (71, 72).

### Crosstalk with cells in the TME

**Figure 3:**
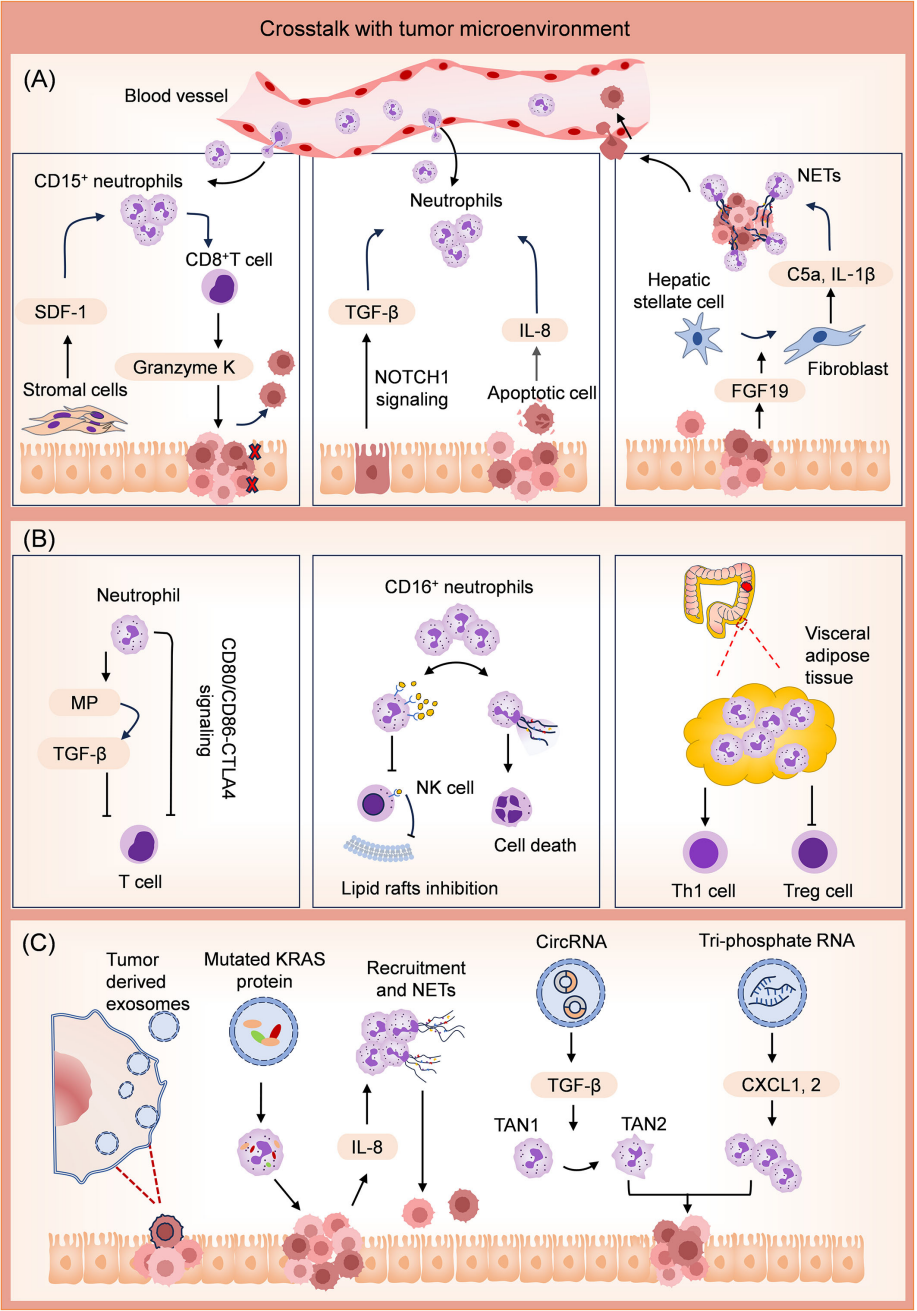
Mechanisms for tumor promotion effects of neutrophils through crosstalk with the TME. **(A)** Crosstalk with several types of cells in TME. The stromal cells facilitate the accumulation of CD15^+^ neutrophils by releasing stromal cell-derived factor-1 (SDF-1). In this case, CD8^+^ T cells produce Granzyme K, diminishing E-cadherin on the intestinal epithelium. Activation of Epithelial NOTCH1 promotes TGFβ-dependent neutrophil recruitment. Apoptotic tumor cells release several neutrophil chemokines and attract neutrophils into the tumor. Tumor cells promote polarization of hepatic stellate cells into inflammatory cancer-associated fibroblasts by releasing fibroblast growth factor 19 (FGF19). These fibroblasts facilitate colonizing tumor cells in the liver by promoting neutrophil infiltration and NET release in liver metastasis niches. **(B)** Crosstalk with other immune cells in TME. Neutrophils suppress the activities of T cells by releasing metalloproteinase (MP) to activate the TGF-β. Neutrophils also promote T-cell exhaustion via CD80/CD86-CTLA4 signaling. CD16^+^ neutrophils competitively inhibit cholesterol intake of NK cells by activating the CD16/TAK1/NF-κB axis. Moreover, CD16^+^ neutrophils can directly induce NK cell death by releasing NETs. In visceral adipose tissue, neutrophils upregulate the level of proinflammatory CD4^+^ Th1 cells and reduce the level of anti-inflammatory Treg cells. The above activities of neutrophils promote the formation of an immunosuppressive TME. **(C)** Crosstalk with the tumor cell-derived exosomes. Exosomes can transfer mutated KRAS protein into neutrophils, stimulate IL-8 secretion of CRC cells, promote neutrophil recruitment, and induce NET formation. Exosomal circRNA promotes the differentiation of TAN1 to TAN2 neutrophils. Exosomal tri-phosphate RNA has been discovered to attract neutrophils through chemokine CXCL1 and CXCL2 secretion.

The TME consists of cellular components, including tumor cells, immune cells, endothelial cells, neurons, etc., as well as extracellular components such as extracellular matrix, extracellular vesicles, and chemokines (73–75). Further study showed that the TME was classified into five distinct states based on the scRNA-seq analysis: immune activation, immune suppression mediated by myeloid or stromal cells, immune exclusion, and immune residence phenotypes (22). Crosstalk between neutrophils and various components in the TME contributes to creating a favorable milieu for tumor initiation and advancement (**Figure 3**).

Communication between various cells in the TME and neutrophils promotes CRC development. The stromal cell-derived factor-1 (CXCL12/SDF-1) facilitates the accumulation of neutrophils expressing high levels of CD15 in CRC tumors, in which case, CD8^+^ T cells produce high levels of Granzyme K, subsequently diminishing E-cadherin on the intestinal epithelium and promotes tumor progression (76). Epithelial NOTCH1 signaling drives metastasis through TGFβ-dependent neutrophil recruitment, which always leads to poor prognostic human CRC subtypes (CMS4 and CRIS-B) (77). Apoptotic tumor cells release a number of neutrophil chemokines, such as IL-8, which strongly attract neutrophils into the tumor (52). The neighboring macrophages facilitate neutrophil activation to accelerate the development of an immunosuppressive TME, potentially contributing to tumor recurrence after chemotherapy-induced apoptosis (35). FGF19 has been identified as a tumorigenic gene in human cancers (78–80). It activates the autocrine of IL-1α through the FGFR4-JAK2-STAT3 pathway, leading to the polarization of hepatic stellate cells into inflammatory tumor-associated fibroblasts (81). These fibroblasts play a role in promoting neutrophil infiltration and NET release in liver metastasis niches by secreting complement C5a and IL‐1β, thereby facilitating the colonization of CRC cells (81). In addition, TANs enhance the migration of CRC cells through CD98hc-xCT via secreting anterior gradient-2 (82).

Neutrophils also influence the state of other immune cells, forming an immunosuppressive TME. T cell‐mediated anti-tumor immune response correlates with favorable disease outcome in cancer immunotherapy. High level of T cell infiltration in CRC is the basis of favorable immunotherapy response (83, 84). Neutrophils from CRC patients suppressed T cell activity via activation of latent TGFβ by neutrophil‐secreted metalloproteinase for creating an immunosuppressive TME *in vitro (* 85). Sui et al. also discovered that in high microsatellite instability (MSI-H) CRC, increased neutrophil infiltration promotes T cell exhaustion by activating CD80/CD86-CTLA4 signaling, which is associated with an immunosuppressive status (86). Activation of the CD16/TAK1/NF-κB axis in CD16^+^ neutrophils up-regulates scavenger receptors for cholesterol intake, thereby enabling these neutrophils to block the formation of NK lipid rafts and transduction of anti-tumor signals by competitively inhibiting cholesterol intake of NK cells. Furthermore, it was found that CD16^+^ neutrophils can directly induce NK cell death by releasing NETs (87). Transcriptome analysis revealed elevated levels of inflammation-related factors and antiapoptotic proteins in VAT neutrophils of obese individuals with CRC. The increase in VAT neutrophils was subsequently accompanied by an upregulation of proinflammatory CD4^+^ Th1 cells and a decrease in anti-inflammatory Treg cells (65).

Exosomes derived from CRC cells are effective vectors for regulating neutrophil activity. Exosomes loading with mutated KRAS protein can transfer mutated KRAS protein into neutrophils and stimulate IL-8 secretion of CRC cells, thereby accelerating neutrophils recruitment and NET formation, eventually leading to CRC deterioration (88). CircPACRGL is an exosomal circular RNA, promoting differentiation of TAN1 to TAN2 neutrophils via miR-142-3p/miR-506-3p-TGF-β axis, further promoting CRC cells proliferation, migration and invasion (89). Exosomal tri-phosphate RNA, derived from CRC stem cells, has been discovered to attract neutrophils through secretion of CXCL1 and CXCL2, as well as to prolong the survival of neutrophils by inducing the expression of IL-1β via the NF-κB pathway (90).

### Others: regulation of DNA repair landscape and angiogenic functions

The direct carcinogenic effect of neutrophils in CRC is associated with generating reactive oxygen species. Excessive H_2_O_2_ from myeloid cells triggers the mutation of genomic DNA and promotes the metaplasia of intestinal epithelial cells. H_2_O_2_ also induces intestinal epithelial cells to secrete cytokines and chemokines through the TNFα autocrine ring to recruit myeloid cells and form positive feedback conducive to tumor development (91).

Neutrophils play a role in modulating the landscape of DNA repair. In low-grade CRC, neutrophils promote deficiency in homologous recombination by down-regulating RAD51 in a Mir-155-dependent manner, thereby impeding tumor growth. However, in advanced CRC, neutrophil-mediated genotoxicity resulting from the accumulation of double-strand breaks induces non-homologous end-joining repair, facilitating tumor survival and proliferation (92).

The angiogenic functions of TANs are primarily associated with two well-established pro-angiogenic factors, VEGF and MMP9 (93–95). The transcriptomic study revealed the enrichment of pathways conducive to angiogenesis and development in TANs. Bioinformatics analysis and functional validation demonstrated that osteopontin and Mmp14, another two angiogenic factors, exhibit the most significant induction effect, effectively regulating endothelial cell activity in CRC. The CRC niche drives pro-angiogenic transcriptional programming in TANs (96), offering a novel strategy for CRC treatment based on the pro-angiogenic properties of TANs.

## Antitumoral role of neutrophils in CRC

**Figure 4:**
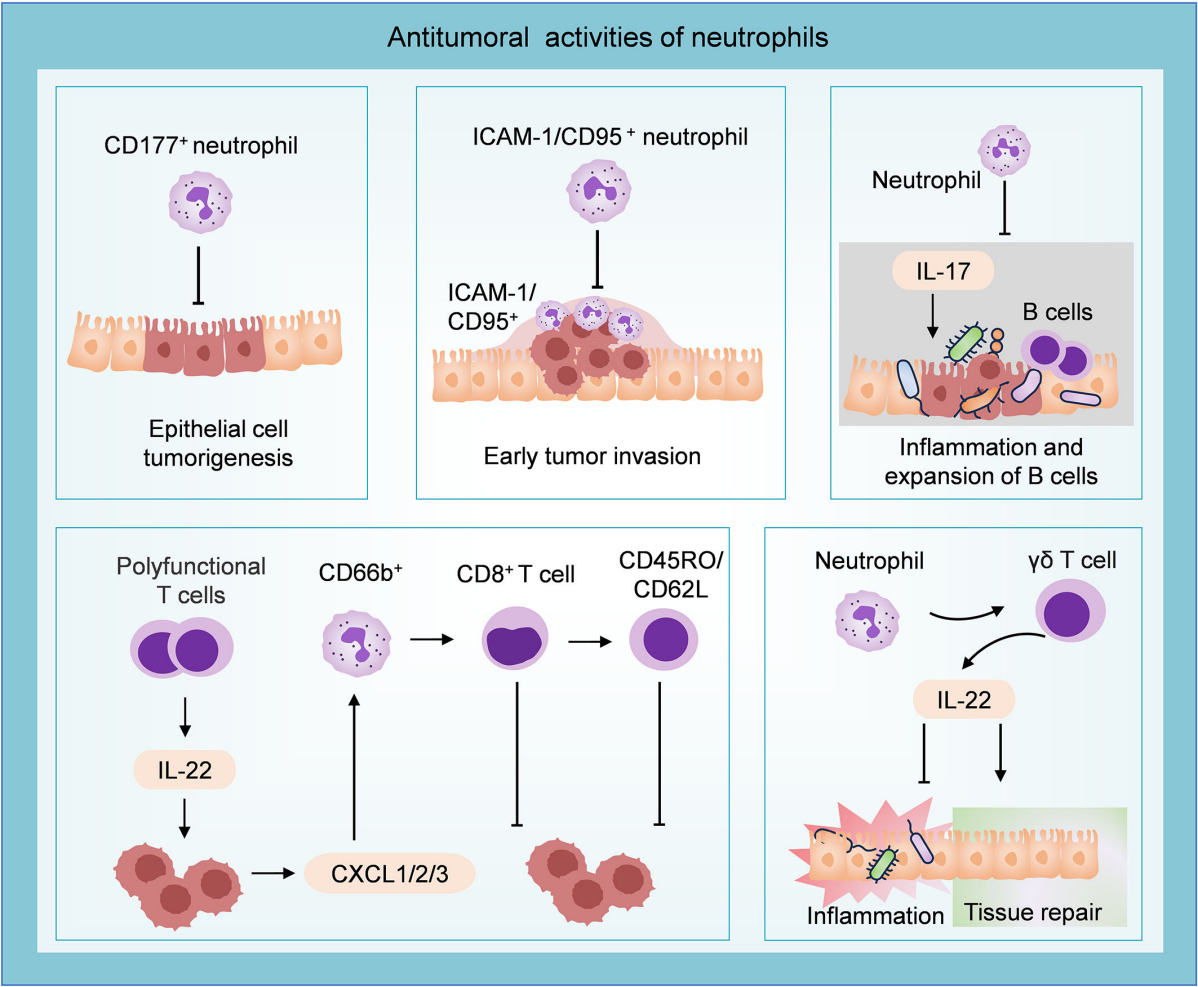
Antitumoral role of neutrophils in CRC. CD177^+^ neutrophils can inhibit epithelial cell tumorigenesis. Neutrophils characterized by ICAM-1 and CD95 infiltrate the invasive edge and restrain the development of early colon tumors. Neutrophils inhibit tumor growth by suppressing IL-17-mediated bacteria-dependent inflammatory response and the expansion of B cells. T cell-derived IL-22 stimulates the secretion of CXCL1/2/3 from tumor cells, then recruits CD66b^+^ neutrophils. These CD66b^+^ neutrophils inhibit tumor progression by augmenting the CD8^+^ T cell response and expanding the “central memory” population. Neutrophils could modulate the gut microbiome-related intestinal inflammation and activate an IL-22-dependent tissue repair approach.

Most studies on the role of neutrophils in tumors have primarily reported their promotion of tumor development; however, they indeed possess anti-tumor functionality in CRC (**Figure 4**). This is intricately associated with the complex TME, tumor stage, and neutrophil heterogeneity.

Neutrophils possess high plasticity and can transit into different anti-tumor roles. Studies have demonstrated a significant increase of CD177^+^ neutrophils in CAC and CRC tissues. Furthermore, patients with high infiltration of CD177^+^ neutrophils (>8 cells/HPF in CRC sections) exhibited improved 5-year overall survival (OS, 80.4% versus 69.3%, *P* = 0.007) and 5-year disease-free survival (DFS, 81.7% versus 71.6%, *P* = 0.010). Subsequent investigation confirmed the ability of CD177^+^ neutrophils to inhibit epithelial cell tumorigenesis (97). Additionally, a study reported that TANs with high levels of ICAM-1 and CD95 displayed an anti-tumor phenotype and were found to infiltrate the invasive edge of early colon tumors, suggesting their potential role in combating the disease at an early stage (98). The strong colocalization of HLA-DR^+^ neutrophils and CD8^+^ T cells in COAD indicates a spatial association between antigen-presenting neutrophils and T cells. HLA-DR^+^ neutrophils can widely trigger T cell activation, antigen reactivity, and cytotoxicity, promoting T cell response and playing a synergistic role in immunotherapy (23). Transcriptome analysis revealed dysregulation of genes involved in antimicrobial and inflammatory processes in neutrophil-deficient colon tumors. The mechanism study demonstrated that polymorphonuclear neutrophils in early colon tumors inhibited the proliferation and aggressiveness of tumor cells by suppressing IL-17-mediated bacteria-dependent inflammatory response and the expansion of B cells in the colon (99).

IL-22 is generated by various immune cells in innate and adaptive immune systems, including group 3 innate lymphoid cells, NK cells, and CD8^+^ T cells (100, 101). Combined survival analysis revealed that the protective performance of IL-22 in CRC relied on the presence of neutrophils. IL-22 can stimulate the secretion of chemokines CXCL1, CXCL2, and CXCL3 by colon tumor cells for recruiting neutrophils. IL-22 augmented the T cell response through the recruitment of beneficial neutrophils (CD66b^+^ cells) and contributed to favorable 5-year OS (58% versus 43%, P = 0.004) (38). Co-cultivation with CD66b^+^ neutrophils enhanced the response of CD8^+^ T cells to the T cell receptors, leading to increased activation and proliferation of CD8^+^ T cells, and expanding the population of cells exhibiting the “central memory” phenotype characterized by CD45RO/CD62L expression (102). A recent study has demonstrated that neutrophil-deficient mice (*Csf3r^−/−^* mice) showed higher susceptibility to carcinogenesis. Neutrophils may confer protection against intestinal inflammation and CAC by modulating the gut microbiome and initiating activation of an γδ T cells-derived IL-22-dependent tissue repair approach (103). IL-22 has been demonstrated to accelerate the development of CRC (104–106), which contradicts the previously mentioned association between IL-22^+^ cell infiltration and improved prognosis. This indicates that the interaction between neutrophils and IL-22 within the established TME exerts multifaceted effects on tumor progression.

## Neutrophils-targeted CRC therapies

Given the multifaceted roles of neutrophils in the pathogenesis and progression of CRC, this review provides several novel insights for developing clinical treatment strategies for CRC patients. Neutrophils are essential components of the host’s defense against infection (24), and their depletion could result in significant immunosuppression. Although existing studies indicate that neutrophils contribute to tumor progression, clinical trials targeting neutrophils remain limited due to concerns about inducing neutropenia. The lack of widely recognized subgroup markers hinders a comprehensive understanding of the heterogeneity of neutrophils, making therapies targeting specific subgroups of neutrophils difficult. Therefore, most research has focused on inhibiting neutrophil-associated molecules known to promote tumor growth and aggressiveness (107). Therefore, we mainly review the clinical studies of key molecules or pathways related to neutrophil function. While not directly depleting neutrophils, these interventions can elucidate the effects of neutrophil-associated targeted or combination therapies on CRC, highlighting their potential to guide clinical therapies.

The importance of the CXCR1/2-IL8 axis in neutrophil chemotaxis and the multiple pro-tumor functions mediated by this axis (35–37) make CXCR1/2-IL8 a desirable therapeutic target. Other classical cytokines such as G-CSF, TGF-β, and VEGF are closely related to the functional status of neutrophils during tumor development (68, 108, 109) and are also important therapeutic targets in CRC treatment. Researchers have found that neutrophils from CRC samples strongly express Bv8/PROK2, while inhibition of G-CSF or Bv8/PROK2 can improve the efficacy of anti-VEGF antibodies and prevent the emergence of drug resistance. The G-CSF/Bv8/PROK2 axis holds potential as a therapeutic target for CRC when combined with anti-VEGF agents (110). In both colitis and CAC models, neutrophil infiltration and pSTAT3 expression increased as the disease progressed. This suggests that neutrophils may promote the transition from colitis to CRC through the JAK/STAT pathway (40). Several clinical trials have explored these targets in depth (**Table 1**).

**Table 1:** Clinical trials based on neutrophil-targeted CRC therapies

Class of target	Agents	Cancer applications	Phase	Clinicaltrails.gov number and status
CXCR1/2 Inhibitor	SX-682	Metastatic CRC	phase II	NCT04599140 (Recruiting)
IL-8 inhibitor	BMS-986253	Stage I-III	phase II	NCT03026140 (Recruiting)
TGF-β pathway inhibitors	Vactosertib	Locally advanced/ Metastatic CRC	Phase Ib	NCT05400122 (Recruiting)
STAT3 pathway inhibitors	TTI-101	Advanced CRC	Phase I	NCT03195699 (Active, not recruiting)
	BBI-608	MSS, refractory CRC	Phase II	NCT03647839 (Completed)
VEGF inhibitors	Tivozanib/ Bevacizumab	Metastatic CRC	phase II	NCT01478594 (Completed)
	Apatinib	Locally advanced dMMR/MSI-H CRC	phase II	NCT04715633 (Active, not recruiting)
G-CSF	Neupogen (filgrastim)	Metastatic CRC	phase II	NCT00541125 (Completed)
		Metastatic CRC	phase II	NCT06504901 (Not yet recruiting)

CXCR1/2, CXC chemokine receptor1/2; CRC, colorectal cancer; IL-8, interleukin-8; TGF-β, transforming growth factor-β; STAT3, signal transducer and activator of transcription 3; VEGF, vascular endothelial growth factor; dMMR/MSI-H CRC, mismatch repair deficiency (dMMR) or high microsatellite instability (MSI-H) CRC; G-CSF, granulocyte colony-stimulating factor.

Studies have shown neutrophils participate in the inhibitory immune microenvironment through the CD80/CD86-CTLA4 pathway, resulting in poor response to immune checkpoint inhibitors (ICIs) in MSI-H patients. In a mouse CRC model, blocking the CD80/CD86-CTLA4 axis under inflammatory conditions improves tumor response to PD-1 blocking (86). The PD1/PD-L1 axis is commonly associated with immune escape and tumor progression. Studies of mouse breast cancer have revealed that tumor cells expressing PD-1 inhibit the cytotoxicity of neutrophils and enhance their metastatic potential through the PD-L1/PD-1 axis. PD-L1^+^ neutrophils are less cytotoxic than PD-L1 neutrophils, and blocking the PD-L1/PD-1 interaction can enhance the anti-tumor cytotoxicity of neutrophils (111). PD-L1^+^ neutrophils in HCC patients can effectively inhibit the proliferation and activation of T cells, and blocking PD-L1 can partially reverse this inhibition (112). In a mouse model of CRC, tumor-derived G-CSF induces neutrophils to express PD-L1 through the STAT3 pathway, and neutrophils promote tumor by inhibiting anti-tumor immunity of NK cells dependent on PD-L1/PD-1. Neutrophil depletion combined with anti-PD-L1 can improve the survival of the mice with CRC (113). These strongly suggest that the PD1/PD-L1 axis is an important therapeutic target under conditions of neutrophil infiltration, and targeting TANs may be an important strategy to prevent PD1/PD-L1-associated tumor immune evasion.

Other functions of TANs are currently under investigation in preclinical studies. PAD4 expressed in neutrophils plays an important role in forming NETs (114). Studies on nude mouse xenograft models have shown that GSK484 (PAD4 inhibitor) can improve the radiosensitivity of CRC, inhibit NET formation, and inhibit tumor growth (115). *F. nucleatum* infection can induce PD-L1 expression by activating the NF- κB/STAT3 signaling in neutrophils, and CX3CR1^+^PD-L1^+^ neutrophils infiltration promotes CRC metastasis and weakens the efficacy of immunotherapy. Treatment with doxycycline eliminated *F. nucleatum* intracellular, thereby reducing CX3CR1^+^PD-L1^+^ neutrophils populations and slowing *F. nucleatum*-promoted tumor growth and metastasis in mice (116). This suggests that the combination of antibiotics and ICIs may benefit such patients.

It should be noted that it has been reported that the combination of anti-TGF-β and anti-PD-L1 therapies may lead to tumor resistance, which has been linked to the upregulation of CR-related metabolic pathways in mice (117). It is recommended that researchers and clinicians should employ a multifaceted approach to assess patient status and develop a precise medical strategy when formulating combination drug regimens targeting TAN.

## Role of neutrophils in prognosis

Multivariate survival analysis found that the high levels (more than 60 per TMA spot) of intratumoral CD66b^+^ neutrophil was an independent risk factor for adverse overall patient survival (hazard ratio [HR]: 2.040; 95% confidence interval [CI]: 1.186–3.843; *P* = 0.010) *(* 118). Multivariate Cox analysis demonstrated that high levels of intra-tumoral CD66b^+^ neutrophils are significantly associated with decreased OS and DFS (119). Interestingly, a separate study reported that co-infiltration of CD66b^+^ neutrophils and CD8^+^ T cells is associated with a more favorable prognosis compared to sole infiltration by CD8^+^ T cells in CRC, potentially due to the complex interplay between these immune cells (102). However, the investigation into the prognostic role of neutrophil infiltration at intra-tumoral subsites in CRC revealed that low levels (semi-quantitatively score, no/sporadic) of neutrophil infiltration in the front of the tumor is an independent prognostic factor for the poor prognosis of patients with early colon cancer (HR: 2.32, 95% CI: 1.45-3.73, *P* <.001) (120). These ostensibly contradictory findings suggest that the prognostic role of neutrophils is multifaceted and may be influenced by their infiltration sites within the TME and their functional status.

The neutrophil-to-lymphocyte ratio (NLR) has been utilized in various types of tumors as a prognostic indicator for poor survival, and it can be employed to inform immune-related treatment strategies and predict clinical outcomes (121–124). Colon cancer patients with a high NLR (> 3.0) exhibit poor 5-year OS (87.0% versus 94.5%, *P =* 0.042) and 5-year relapse-free survival (RFS) (77.9% versus 87.8%, P = 0.032) (125). The high NLR serves as a crucial prognostic indicator for advanced colon cancer, particularly in the case of left-sided colon cancer (5-year OS: 86.4% versus 95.2%, P = 0.014; 5-year RFS:79.2% versus 87.3%, P = 0.047) (125). Multivariate analysis showed that increased NLR (> 5.0) in CRC liver metastasis was associated with poor 5-year OS (27% versus 47%, P < 0.01) and 5-year DFS (6% versus 37%, *P* < 0.01) (126). Low baseline NLR (<5.11, HR: 0.42, 95% CI: 0.21-0.84, *P* = 0.014) and early NLR reduction after two courses of immunotherapy (HR: 0.33, 95% CI: 0.18-0.61, *P* < 0.001) were significantly associated with better outcomes in CRC patients. Among them, patients with a low baseline NLR and an early decline in NLR exhibited the longest median OS (n = 53, median OS = 29.63 months) (127). Further analysis showed that a combination of NLR and tumor mutation burden provided additional predictive capacity (127). Currently, several clinical studies have investigated the practical clinical significance, including the exploration of NLR (NCT05673343, NCT06495827), combination of NLR with C-reactive Protein (NCT05129046), and neutrophils-circulating tumor cells analysis (NCT05793775) in diagnosis and prediction of advanced CRC.

Further research on neutrophil-related genes in CRC patients identified 17 genes significantly associated with OS. Based on these 17 genes, a prognostic risk score (PRS) system was developed. Patients in the high-PRS group were more likely to experience tumor recurrence and metastasis. However, high-PRS group patients showed improved response to immunotherapy, possibly due to increased tumor mutation burden and MSI. Therefore, the PRS can serve as a prognostic model for guiding individualized treatment for colon cancer patients (128).

Neutrophil infiltration can also predict the response of CRC patients to the drug therapies. A study revealed that neutrophil infiltration increases in stage I-III CRC but decreases significantly in stage IV, possibly indicating immune escape in advanced disease. In stage III CRC, high TAN infiltration is linked to a favorable response to a 5-fluorouracil-based chemotherapy regimen (better DFS, HR: 0.42, *p* = 0.01), suggesting that evaluating TAN infiltration may help identify patients who would benefit from this therapy (129). Due to neutrophil-associated immunosuppression, local inflammatory conditions in MSI-H CRC can hinder tumor response to ICIs, potentially leading to ICI resistance. It has been demonstrated that inflammation infiltration and high NLR (> 3.0) are clinical features of adverse ICI reactions in MSI-H CRC (86).

## Conclusion and prospects

CRC is characterized by chronic inflammation, which is thought to function in tumor progression and metastasis. The neutrophils are vital for forming the inflammatory TME via the action of NETs, their interaction with the gut microbiota, and crosstalk with other cells such as tumor cells, T cells, and macrophages. However, neutrophils exhibit a dual role in cancers, which may be achieved through two mechanisms (13, 18, 23): (1) Neutrophils themselves possess heterogeneity, with different subtypes performing distinct functions; (2) Neutrophils undergo reprogramming within the TME. The function of neutrophils might result from a combination of these two mechanisms, which requires more evidence.

In CRC, TANs play a crucial role in the immune evasion mechanism during therapy by shaping an immunosuppressive microenvironment and promoting tumor resistance to immune checkpoint therapy through inflammation response. Targeting the molecules of the pathways involved in TANs-mediated tumor progression and therapeutic resistance can lead to the development of multiple synergistic therapeutic strategies. Several approaches have been explored for targeting TANs (107, 130): (1) Inhibition of TANs enrichment, proliferation, and polarization (131); (2) Suppression of neutrophil-induced immune escape, especially enhancing reaction of tumor cells to ICIs (132); (3) Inhibition of other key molecules associated with neutrophil function (133); (4) Utilization of neutrophils as drug delivery carriers based on their innate inflammatory response sensitivity and ability to cross physical barriers (134, 135). These pathways alone or in combination may bring breakthroughs in treating CRC.

The anti-tumor effect of neutrophils in CRC should not be disregarded, and it is worth considering amplifying the antagonistic effect of specific neutrophils subtypes to control the progression of CRC. Furthermore, a study has demonstrated that in successful immunotherapy for mice with lung tumors, neutrophils characterized by interferon-stimulated genes rapidly accumulate in tumor tissues and exhibit an anti-tumor phenotype (136). Another perspective suggests that T cells can eliminate tumor cells with high antigen density, while neutrophils kill tumor antigen escape variants, and immunotherapy with the combination of the two can eliminate highly heterogeneous tumors (137). Therefore, immunotherapy that induces anti-tumor T cells can be combined with therapy that optimizes the anti-tumor function of neutrophils, which may lead to more durable tumor control following treatment.

Neutrophils remain technical challenges in study due to their fragility, short lifespan, and low RNA content. Although mouse models are commonly employed for studying human tumors, they do not accurately reflect the *in vivo* conditions. Research has demonstrated that human neutrophils selectively release active neutrophilic elastase to eliminate various cancer cells while sparing non-cancer cells; however, this property is absent in mouse neutrophils (138). Therefore, there is a pressing need to develop reliable research techniques for studying human-derived TANs to obtain more credible insights into the mechanisms by which TANs regulate cancers. To simulate the *in vivo* environment more accurately, we propose that organ-on-a-chip represents an advanced model for *in vitro* research of neutrophils. It is feasible to recreate a TME on a microfluidic chip, presenting significant application potential (139). Intravital microscopy (IVM) combined with fluorescent neutrophil reporter mice enabled real-time visualization of neutrophil dynamics in tumors. Commonly used *in vivo* imaging techniques include confocal and multiphoton microscopy. Imaging techniques can broaden our understanding of how neutrophils are involved in cancer development, providing a powerful tool for neutrophilic research (140). The microbiome is involved in the establishment of the TME. The microbiome can trigger the immune system response directly or influence the functional state of immune cells via metabolic processes and metabolites (141). The combination of microbiome and immunotherapy is also a direction that researchers should keep exploring (142). Proteomic, biomechanical, and functional analyses can elucidate neutrophil heterogeneity in systemic lupus erythematosus, offering another possible method to investigate neutrophil heterogeneity in CRC (143).

In recent years, scRNA-seq technology has been utilized for the analysis of TAN characteristics in tumors, revealing the heterogeneous subtypes within the TME and elucidating the role of each subtype in tumor development and response to therapy (21, 22). Despite extensive studies on consensus molecular subtype classification of CRC (144–146), there is a lack of comprehensive analysis on TAN subtypes using scRNA-seq, which could provide valuable insights into assessing CRC prognosis and treatment based on TAN heterogeneity. The following directions can be considered for scRNA-seq of neutrophils from CRC patients: (1) Identify the TAN subtypes in human CRC; (2) Characterize molecular markers specific to each TAN subtype, with a focus on the pro-tumor subtype; (3) Investigate the differentiation pathways that lead to the development of pro-tumor neutrophil subtype; (4) Examine the metabolic reprogramming in tumor-promoting TANs and evaluate how these metabolic changes significantly influence immune cell function; (5) Analyze the interactions between specific TAN subtypes and other immune cells such as T cells and NK cells; (6) Based on these findings, explore strategies to block key molecules involved in the differentiation pathway of pro-tumor neutrophils. For instance, inhibiting upstream regulators and disrupting the active metabolic programs of TANs can suppress the pro-tumor functions and immunosuppressive activities of neutrophils, thereby identifying potential TAN-related therapeutic targets for CRC.

ScRNA-seq research of neutrophils poses several challenges due to their biological characteristics, functional heterogeneity, and technical limitations. Neutrophils are fragile and short-lived cells (with a half-life of 7 to 10 hours) (147) and have low mRNA content(0.33 μg per million cells) (148), challenging the isolation of neutrophils and the subsequent extraction of RNA. In a recent study, the lack of crucial biomarkers could restrict the accuracy of identifying the cells among neutrophil subsets with similar transcriptomic characteristics (149). ScRNA-seq is also insufficient to fully capture the spatiotemporal characteristics of neutrophils. The transcriptomes of neutrophils can vary depending on their activation states and microenvironment, making it difficult to comprehensively describe their transcriptional landscapes at the single-cell level (21). Existing datasets lack spatial information regarding tissue distribution and cell-cell interactions; fortunately, rapidly evolving spatial profiling technologies may address these limitations (150). Other inherent limitations of scRNA-seq technology should not be overlooked, including its limited sensitivity, scale, accuracy, and noise introduced by single-cell RNA preamplification (151). Overcoming these challenges requires a robust experimental design, advanced computational methodologies, and a profound understanding of neutrophil characteristics.
